# Global Pattern and Trends in Penile Cancer Incidence: Population-Based Study

**DOI:** 10.2196/34874

**Published:** 2022-07-06

**Authors:** Leiwen Fu, Tian Tian, Kai Yao, Xiang-Feng Chen, Ganfeng Luo, Yanxiao Gao, Yi-Fan Lin, Bingyi Wang, Yinghui Sun, Weiran Zheng, Peiyang Li, Yuewei Zhan, Christopher K Fairley, Andrew Grulich, Huachun Zou

**Affiliations:** 1 School of Public Health (Shenzhen) Sun Yat-sen University Guangzhou, Guangdong China; 2 Department of Urology Sun Yat-sen University Cancer Center State Key Laboratory of Oncology in South China, Collaborative Innovation Center for Cancer Medicine Guangzhou China; 3 Center for Reproductive Medicine Ren Ji Hospital Shanghai Jiao Tong University Shanghai China; 4 Shanghai Key Laboratory for Assisted Reproduction and Reproductive Genetics Shanghai China; 5 Shanghai Human Sperm Bank Shanghai China; 6 Central Clinical School Monash University Melbourne Australia; 7 Melbourne Sexual Health Centre Alfred Health Melbourne Australia; 8 Kirby Institute University of New South Wales Sydney Australia

**Keywords:** global burden, penile cancer, incidence, average annual percentage change, epidemiology

## Abstract

**Background:**

Penile cancer is a relatively rare genital malignancy whose incidence and mortality are rising in many countries.

**Objective:**

This study aims to assess the recent incidence and mortality patterns and incidence trends of penile cancer.

**Methods:**

The age-standardized incidence and mortality rates (ASIR and ASMR, respectively) of penile cancer in 2020 were estimated from the Global Cancer Registries (GLOBOCAN) database. Incidence trends of penile cancer from 1973 to 2012 were assessed in 44 populations from 43 countries using the Cancer Incidence in Five Continents plus (CI5*plus*) and the Nordic Cancer Registries (NORDCAN) databases. Average annual percentage change was calculated to quantify trends in ASIR using joinpoint regression.

**Results:**

Globally, the estimated ASIR and ASMR of penile cancer were 0.80 (per 100,000) and 0.29 (per 100,000) in 2020, equating to 36,068 new cases and 13,211 deaths in 2020, respectively. There was no significant correlation between the ASIR (*P*=.05) or ASMR (*P*=.90) and Human Development Index. In addition, 15 countries saw increasing ASIR for penile cancer, 13 of which were from Europe (United Kingdom, Lithuania, Norway, Estonia, Finland, Sweden, Cyprus, Netherlands, Italy, Croatia, Slovakia, Russia, and the Czech), and 2 from Asia (China and Israel).

**Conclusions:**

Although the developing countries still bear the higher incidence and mortality of penile cancer, the incidence is on the rise in most European countries. To mitigate the disease burden resulting from penile cancer, measures to lower the risk for penile cancers, including improving penile hygiene and male human papillomavirus vaccination, may be warranted.

## Introduction

Penile cancer is rare and can occur anywhere on the penis, although most cases arise from the squamous epithelium of glans, coronal sulcus, and prepuce or foreskin. About 95% of penile cancer is classified as squamous cell carcinoma but penile cancer also includes sarcoma, melanoma, and basal cell carcinoma [1,2]. The estimated age-standardized incidence of penile cancer worldwide was 0.80 per 100,000 person-years in 2018, and the incidence is predicted to increase by more than 56% by 2040, according to the Global Cancer Registries (GLOBOCAN) Cancer Tomorrow prediction tool [3]. The change can be largely attributed to the increasing aging of the population, as penile cancer mostly affects older men with a peak in incidence in the sixth decade [4]. In certain Asian, African, and South American countries, the incidence of penile cancer constitutes up to 10% of malignancies in men [4,5]. The 5-year survival rate of penile cancer is about 65% but greater in countries with greater access to treatment [6]. The treatments for penile cancer can be disfiguring and affects the patient’s quality of life and sexual function [4,7].

There are many causes of penile cancer. Factors that increase the risk include phimosis, poor personal hygiene, and persistent high-risk human papillomavirus (HPV) infection [8,9]. Phimosis can lead to poor sanitation under the preputial skin and accumulation of smegma, which has been proved to be carcinogenic in animals [4,10-12]. Phimosis may also aggravate balanitis and dermatitis, and is related to the development of aggressive penile malignancies [4,13,14]. Uncircumcised men with poor genital hygiene, even without phimosis, may also have retention of microorganisms and secretions [12,15,16]. A meta-analysis study reported that the pooled detection rate of HPV DNA among penile cancer cases was 50.8% [17]. HPV-16, HPV-6, and HPV-18 are the most common types involved [17-19]. Precancerous lesions associated with HPV infection increase the risk of invasive penile cancer, such as Bowen disease, erythroplasia of Queyrat, and bowenoid papulosis [4]. Furthermore, lack of circumcision, tobacco use, ultraviolet A phototherapy, lichen sclerosis, penile trauma, and low socioeconomic status are also found to be associated with penile cancer [8]. The incidence of penile cancer is negatively correlated with the Human Development Index (HDI) [20].

The incidence of penile cancer has been increasing in many areas in the past few decades [21-23]. There was a 21% increase, from 1.1 to 1.3 per 100,000, in penile cancer incidence in England between 1979 and 2009 [24]; in Norway, the incidence of penile cancer increased from 0.6 to 0.9 per 100,000 between 1956 and 2015 [21]; the incidence of penile cancer in Germany increased from 1.2 per 100,000 in 1961 to 1.8 per 100,000 in 2012 [22]. While previous studies have focused on the incidence trend of penile cancer in specific regions and populations, few reports are available on global patterns and long-term trends in the burden of penile cancer. Understanding the epidemiology of penile cancer can help shed light on factors underlying changing trends.

We aimed to examine the geographical variations in incidence and mortality patterns of penile cancer among 185 countries in 2020 and the long-term incidence trends in 43 countries with 44 populations during the period between1973 and 2012. Our objective is to inform future research and assist policymakers in adopting sound cancer control initiatives.

## Methods

### Data Source

The estimated data were extracted from the GLOBOCAN Database from the International Agency for Research on Cancer (IARC) [25] to assess the global burden of penile cancer in 185 countries and regions in 2020 [26]. The population-based penile cancer incidence data, with the requirement of at least 15 consecutive years of data, were from Cancer Incidence in 5 Continents (CI5) volumes [27], CI5*plus* [28], and the Nordic Cancer Registries (NORDCAN) database [29]. The quality of the data sources used in this paper has been evaluated in previous studies to assess the incidence trends of other cancers [30-32]. The CI5*plus* database contains updated annual incidence rates for 124 selected populations from 108 cancer registries published in CI5, representing 43 countries, for the period from 1973 to 2012 [32]. The NORDCAN database and program include detailed information and results on cancer incidence, mortality, and prevalence in each of the Nordic countries over 5 decades.

Four levels of HDI were used to further assess the cancer burden according to a binary proxy of development (low and medium HDI vs high and very high HDI) in GLOBOCAN 2020. The incidence data of Denmark, Finland, Iceland, Norway, and Sweden were extracted from the NORDCAN database for the years 1953-2016 [33]. The incidence data of Australia, Croatia, Czech Republic, New Zealand, and Russia were supplemented by their corresponding official national cancer data (Table 1) [34-38]. For volume XI, years 2008-2012 included plotting the overall age-standardized rate by country. Overall, incidence trends were evaluated for 44 populations from 43 countries. As many as 24 out of 43 countries were nationally representative and the representativeness of data in the remaining 19 countries has been verified in previous studies [39,40].

### Statistical Analysis

The age-standardized rates were calculated using the World standard population [41]. Trends in incidence are shown with smoothed lines on fitting locally weighted regression (LOWESS) curves, and joinpoint regression (Joinpoint regression program 4.9.0.0, available through the Surveillance Research Program of the US National Cancer Institute) was used to assess temporal trends, which involves fitting a series of joined straight lines to age-standardized incidence rates (ASIRs) trends [42]. Changes in annual incidence rates of penile cancer were calculated as an annual percentage change (APC) in each segment. The joinpoint analysis provided the average annual percentage change (AAPC). To comprehensively estimate the magnitude and direction of trends, we calculated the AAPC and the corresponding 95% CI for the last available 15 years (1998-2012) and those available during the completed period in each country from the database. Correlation analysis was used to test the correlation between the ASIR or age-standardized mortality rate (ASMR) and HDI. All statistical analyses were done using R software 3.6.0 (R Core Team).

## Results

### Prediction of Incidence and Mortality Patterns in 2020

**Figure 1:**
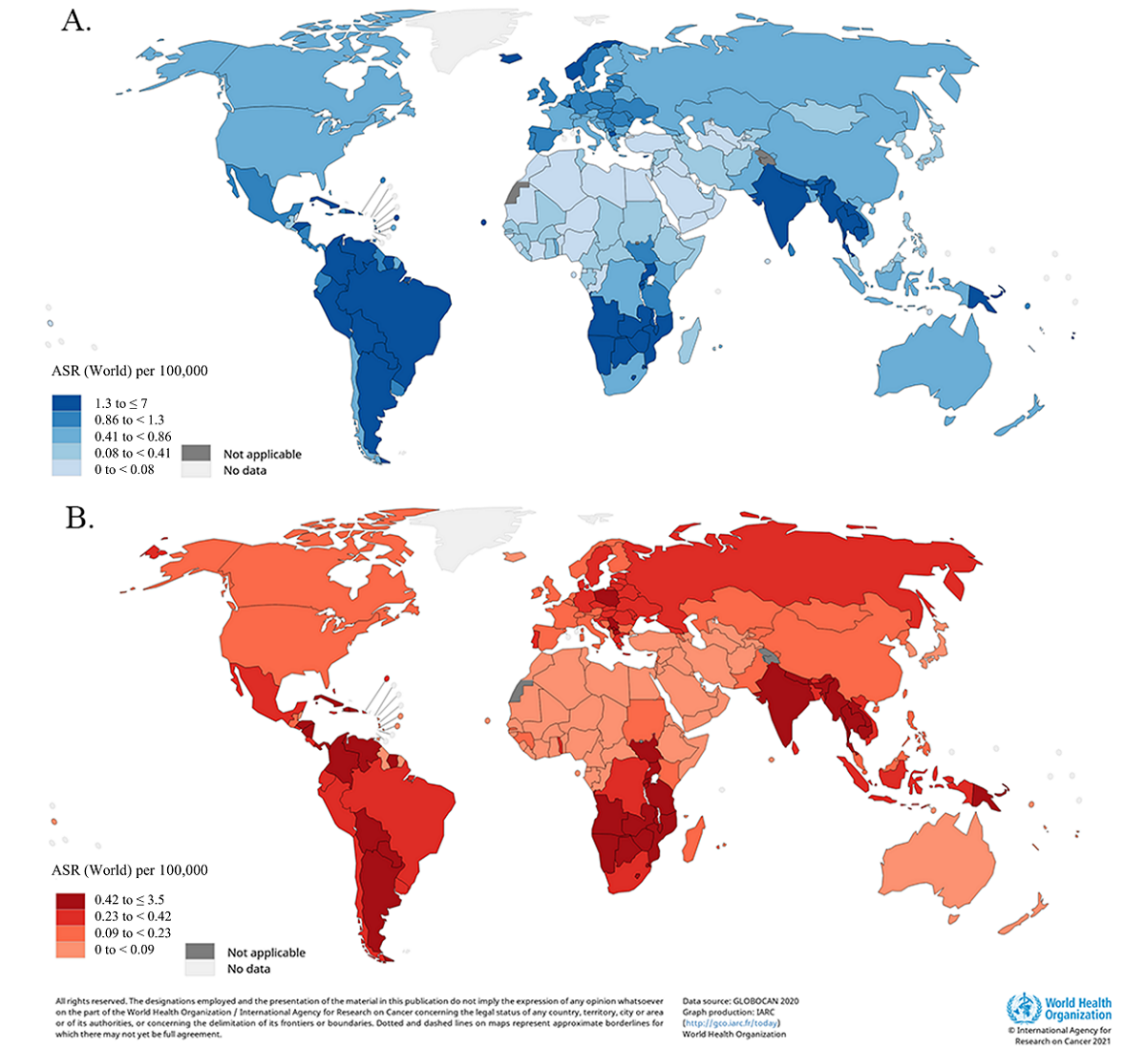
Estimated age-standardized incidence rate (world) in 2020 for penile cancer (A) and estimated age-standardized mortality rate (world) in 2020 for penile cancer (B). (GLOBOCAN 2020 [25]). Data obtained from GLOBOCAN 2020. Map produced by the IARC and WHO. [26].

The global estimated ASIR of penile cancer was 0.8 (per 100,000) in 2020, with estimates indicating 36,068 newly diagnosed cases (Figure 1A and Multimedia Appendix 1). The ASIR of penile cancer varied among 5 continents, with higher ASIRs being observed in Southern Africa, South Asia, and South America. In 2020, the largest number of incident cases was estimated to have occurred in India (n=16,677), China (n=4628), and Brazil (n=1658). The highest ASIRs were found in Eswatini (7.0 per 100,000), Uganda (4.6 per 100,000), and Botswana (4.4 per 100,000), while the lowest were mostly concentrated in countries in Northern Africa, such as Nigeria and Libya (less than 0.01 per 100,000).

Estimates suggest that 13,211 men with penile cancer died in 2020 globally, corresponding to an ASMR of 0.29 cases per 100,000 (Figure 1B and Multimedia Appendix 2). Geographical patterns of ASMR were similar to those of ASIR, and the highest penile cancer ASMRs were noted in Eswatini (3.5 per 100,000) and Uganda (2.4 per 100,000). The largest number of deaths occurred in India (n=4760), China (n=1565), and Brazil (n=539) in 2020.

**Figure 2:**
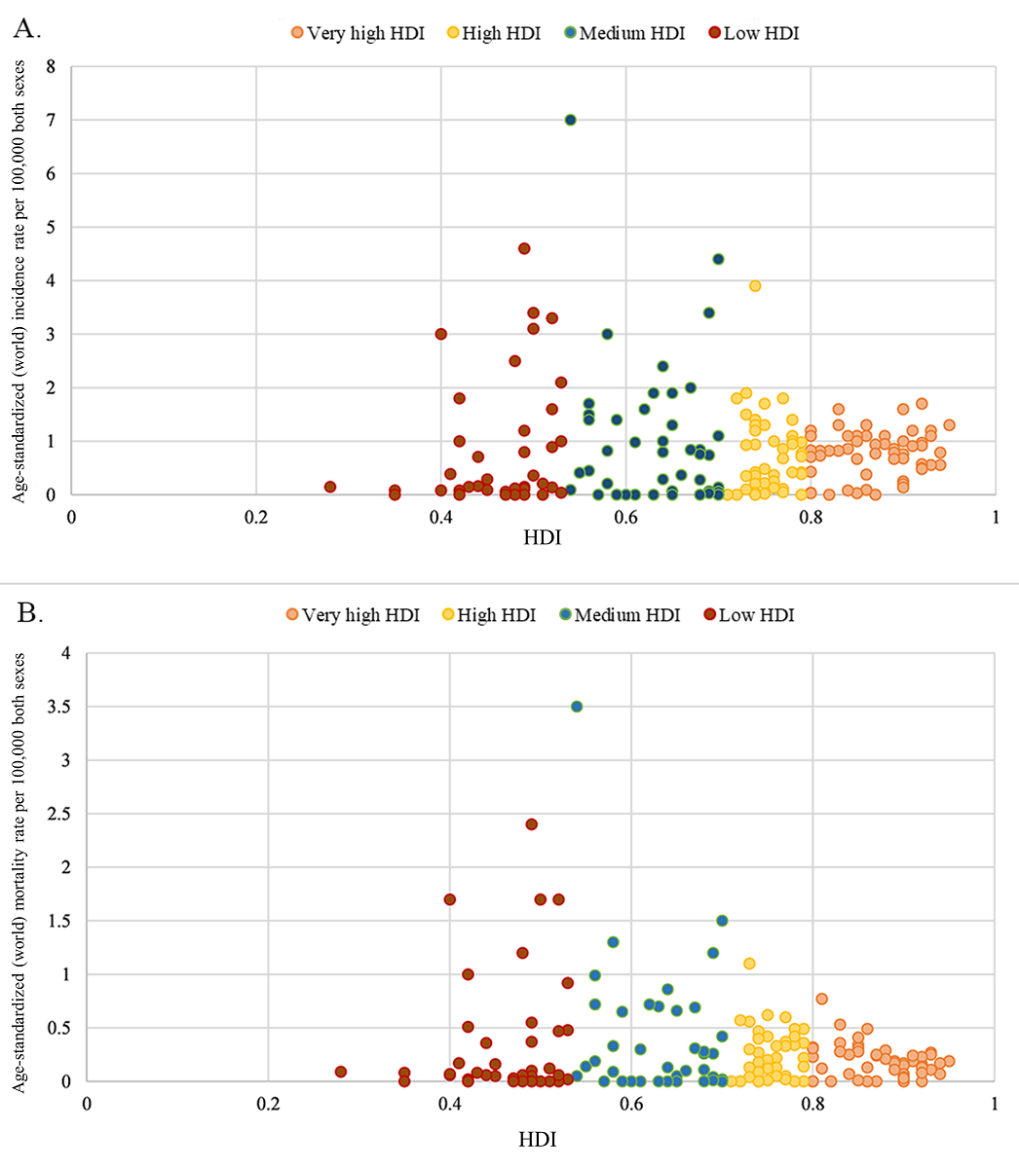
Distribution between (A) age-standardized incidence and (B) mortality rates of penile cancer and HDI (GLOBOCAN 2020). HDI: human development index.

Both ASIR and ASMR in low- and middle-income countries were nearly twice as those in high-income countries (Multimedia Appendices 1 and 2). Low- and middle-income countries accounted for 40.20% (14,499/36,068) of the incidence and 47.73% (6305/13,211) of the deaths from penile cancer globally, respectively. There was no significant correlation between the ASIR or ASMR and HDI (*ρ*=1.43, *P*=.05; *ρ*=−0.01, *P*=.90; Figure 2).

### Incidence Rates in 2008-2012

**Figure 3:**
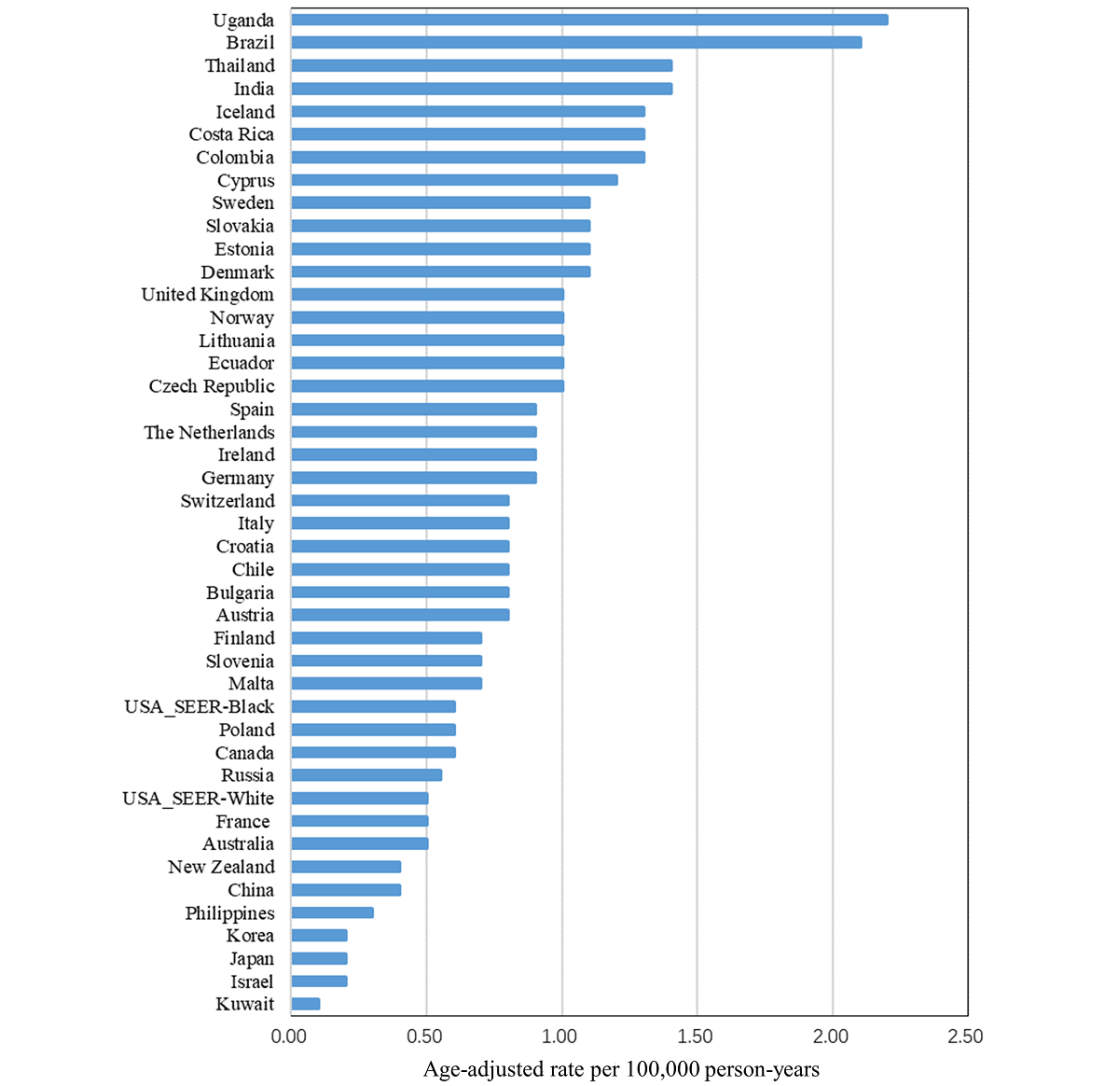
Age-standardized (world standard population) rates of penile cancer incidence, 2008-2012. SEER: Surveillance, Epidemiology, and End Results Program.

Among 44 populations from 43 countries included in the analysis, the highest ASIR of penile cancer between 2008 and 2012 was in Uganda (2.2 per 100,000), followed by Brazil (2.1 per 100,000), Thailand (1.4 per 100,000), and India (1.4 per 100,000) (Figure 3). The lowest ASIR was in Kuwait (0.1 per 100,000) and ASIRs were less than 0.5 per 100,000 in East Asia and West Asia.

### Trends in Incidence

**Figure 4:**
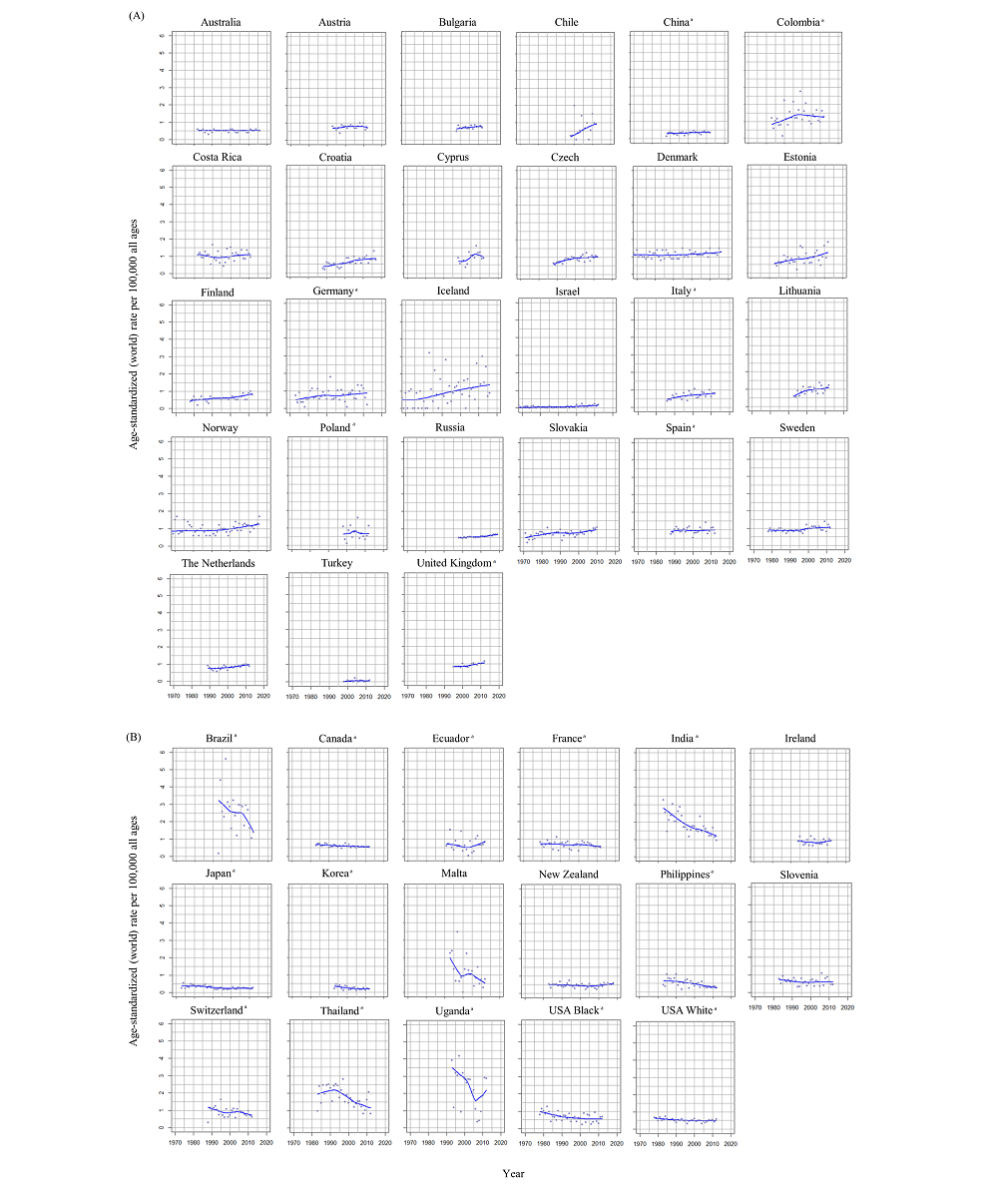
Age-standardized incidence rates of penile cancer. (A) Trends in penile cancer incidence increasing. (B) Trends in penile cancer incidence decreasing. ^a^Regional data.

The trends in the ASIR of penile cancer between one year and another in 44 populations from 43 countries are displayed in Figure 4. Limited data in some populations meant we could calculate the AAPCs of penile cancer only in 40 populations from 39 countries (Table 1). The largest increase in ASIR was in Israel (AAPC 7.2, 95% CI 3.4 to 11.1; *P*=.001), followed by Cyprus (4.6, 95% CI 0.2 to 9.1; *P*=.04), Croatia (3.6, 95% CI 2.2 to 5.0; *P*<.001), and Lithuania (2.6, 95% CI 0.8 to 4.4; *P*=.007). ASIRs for penile cancer have significantly increased in 15 populations of which 7 were from Northern Europe (United Kingdom, *P*<.001; Lithuania, *P=*.007; Norway, *P=*.002; Estonia, *P=*.02; Finland, *P=*.001; Sweden, *P=*.006; and Cyprus, *P=*.04). In Uganda, the ASIR trend of penile cancer showed a rapid increase between 2007 and 2012. The corresponding APC was 53.3 (95% CI 12.4 to 109.0; *P*=.01).

ASIRs of penile cancer in 5 out of 40 populations, including 3 from Northern America and 2 from Asia, significantly decreased. These decreases were in the Philippines (–2.9, 95% CI –4.5 to –1.2; *P*=.002), India (–2.5, 95% CI –3.4 to –1.6; *P*<.001), the USA White (–1.9, 95% CI –3.1 to –0.6; *P*=.006), the USA Black (–0.8, 95% CI –1.4 to –0.3; *P*=.006), and Canada (–0.7, 95% CI –1.2 to –0.2; *P*=.004). In Thailand, the ASIR of penile cancer decreased between 1988 and 2012. The corresponding APC was –3.4 (95% CI –4.9 to –1.8; *P*<.001).

**Figure 5:**
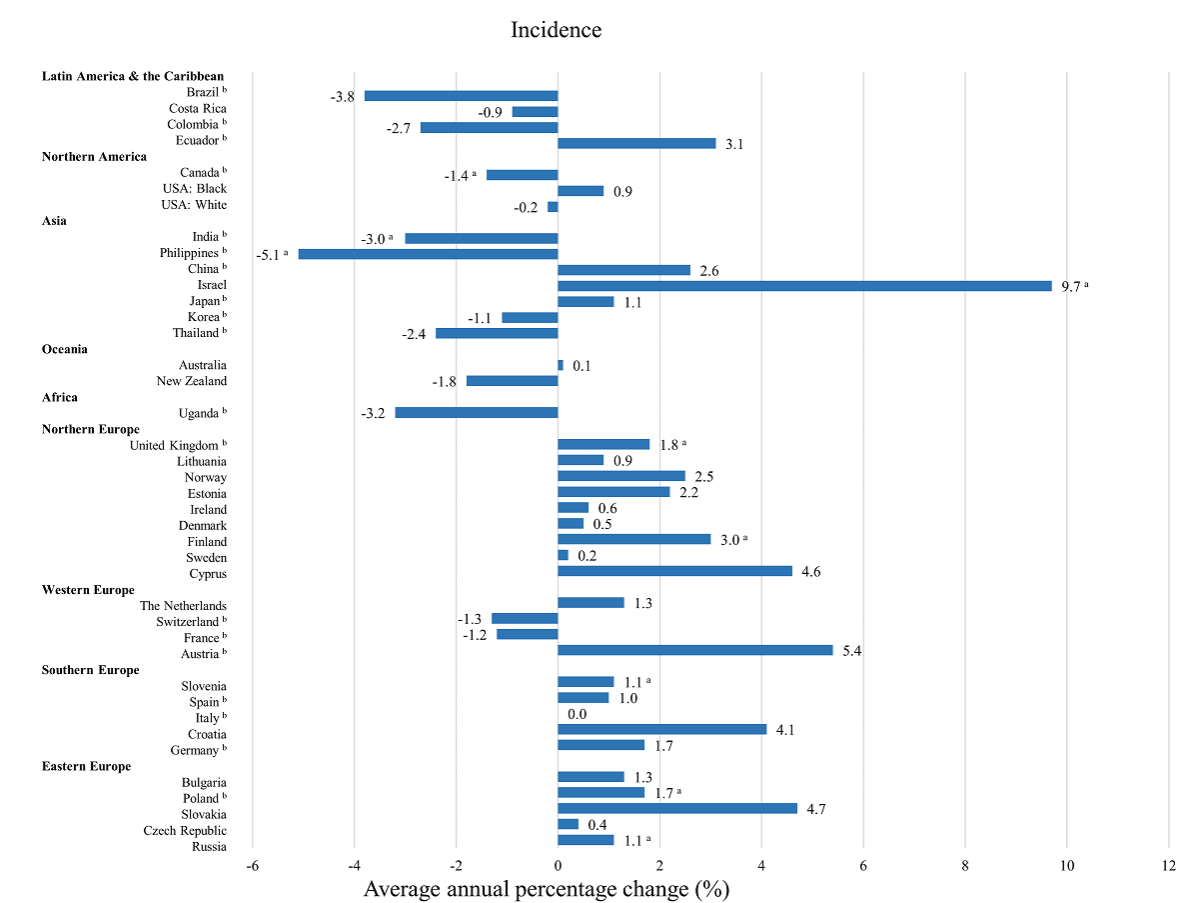
Average annual percentage change (AAPC) of penile cancer incidence in the recent 15 years (1998-2012). ^a^statistically significant; ^b^regional data (incidence).

ASIRs of penile cancer in other parts of Europe, apart from France and Switzerland, increased over the 15-year period (1998-2012; Figure 5). These increases were significant in Russia (AAPC 1.1, 95% CI 0.3 to 1.9; *P*=.01), United Kingdom (1.8, 95% CI 0.8 to 2.8; *P*=.002), Finland (3.0, 95% CI 0.2 to 5.9; *P*=.006), Croatia (4.1, 95% CI 0.7 to 7.5; *P*=.02), Slovakia (4.7, 95% CI 3.2 to 6.2; *P*<.001), and Cyprus (4.6, 95% CI 0.2 to 9.1; *P*=.04). In Asia, significant increases were only observed in Israel (9.7, 95% CI 1.6 to 18.5; *P*=.02), whereas ASIR decreased in India (–3.0, 95% CI –5.2 to –0.8; *P*=.01) and the Philippines (–5.1, 95% CI –8.0 to –2.2; *P*=.003).

**Table 1:** International variation in carcinoma of penis incidence rates.

Countries	Registries	Database source	Period	APC^a^	AAPC (%)^b^	AAPC (95% CI)
Austria	National	[43]	1993-2012		0.9	–0.7 to 2.6
Australia	National	[34]	1982-2016		0.1	–0.5 to 0.6
Brazil	Goiania	CI5*plus*^c^	1993-2012		–0.0	–5.8 to 6.1
Bulgaria	National	CI5*plus*	1998-2012		1.3	–0.5 to 3.1
Canada	Alberta, British Columbia, Manitoba, Newfoundland, Nova Scotia, Ontario, Prince Edward Island, Saskatchewan	CI5*plus*	1983-2012		–0.7^d^	–1.2 to –0.2
China	Shanghai	CI5*plus*	1988-2012		1.6^d^	0.1 to 3.2
Colombia	Cali	CI5*plus*	1983-2012		1.8	–0.3 to 4.0
Costa Rica	National	CI5*plus*	1982-2011		0.2	–1.1 to 1.6
Croatia	National	[44]	1988-2017		3.6^d^	2.2 to 5.0
Cyprus	National	CI5*plus*	1998-2012		4.6^d^	0.2 to 9.1
Czech Republic	National	[45]	1977-2018		2.0^d^	1.6 to 2.4
Denmark	National	NORDCAN^e^ database	1953-2016		0.1	–0.1 to 0.4
Ecuador	Quito	CI5*plus*	1991-2011		–1.3	–6.6 to 4.4
Estonia	National	CI5*plus*	1983-2012		2.2^d^	0.4 to 4.0
Finland	National	NORDCAN database	1953-1982	–1.9^d^		–3.1 to –0.7
		NORDCAN database	1982-2015	1.7^d^		0.7 to 2.7
		NORDCAN database	1953-2015		0.0	–0.4 to 0.5
France	Bas-Rhin, Calvados, Doubs, Isere	CI5*plus*	1979-2012		–0.5	–1.5 to 0.5
Germany	Saarland	CI5*plus*	1973-2012		0.9	–0.9 to 2.7
India	Chennai	CI5*plus*	1983-2012		–2.5^d^	–3.4 to –1.6
Ireland	National	CI5*plus*	1994-2012		–0.1	–1.8 to 1.6
Israel	National	CI5*plus*	1988-2012		7.2^d^	3.4 to 11.1
Italy	Biella, Naples, Parma, Romagna, Ragusa	CI5*plus*	1986-2012		2^d^	0.7 to 3.2
Japan	Miyagi Prefecture, Nagasaki, Osaka Prefecture	CI5*plus*	1973-1986	1.4		–1.9 to 4.8
		CI5*plus*	1986-1992	–10.6		–22.3 to 2.9
		CI5*plus*	1992-2012	1		–0.7 to 2.8
		CI5*plus*	1973-2012		–0.7	–3.2 to 1.8
Korea	Busan, Seoul, Gwangju, Incheon	CI5*plus*	1993-2012		–3.1	–6.1 to 0.1
Lithuania	National	CI5*plus*	1993-2012		2.6^d^	0.8 to 4.4
The Netherlands	National	CI5*plus*	1989-2012		1.3^d^	0.5 to 2.1
New Zealand	National	[37]	1983-2009	–1.3^d^		–2.6 to –0.1
		[37]	2009-2018	6.1		–0.3 to 12.9
		[37]	1983-2018		0.5	–1.2 to 2.3
Norway	National	NORDCAN database	1953-2016		0.6^d^	0.2 to 1.0
Philippines	Manila	CI5*plus*	1983-2012		–2.9^d^	–4.5 to –1.2
Poland	Kielce	CI5*plus*	1998-2012		1.7	–6.6 to 10.9
Russia	National	[38]	1998-2019		1.6^d^	1.1 to 2.0
Slovakia	National	CI5*plus*	1971-2012		1.4^d^	0.6 to 2.1
Slovenia	National	CI5*plus*	1983-2012		–0.5	–1.9 to 0.9
Spain	Basque, Tarragona, Granada, Girona	CI5*plus*	1988-2012		0.2	–0.9 to 1.4
Sweden	National	NORDCAN database	1960-1989	–0.5		–1.1 to 0.1
		NORDCAN database	1989-2016	1.0^d^		0.3 to 1.6
		NORDCAN database	1960-2016		0.2	–0.1 to 0.4
Switzerland	Geneva, Neuchatel, Vaud	CI5*plus*	1988-2012		–0.2	–2.3 to 1.9
Thailand	Chiang Mai	CI5*plus*	1983-1988	13.8		–4.1 to 35.0
		CI5*plus*	1988-2012	–3.4^d^		–4.9 to –1.8
		CI5*plus*	1983-2012		–0.6	–3.6 to 2.5
Uganda	Kampala	CI5*plus*	1993-2004	1.2		–7.9 to 11.1
		CI5*plus*	2004-2007	–46.8		–86.7 to 112.9
		CI5*plus*	2007-2012	53.3^d^		12.4 to 109
		CI5*plus*	1993-2012		2	–17.8 to 26.5
United Kingdom	East England, East Midlands, London, Northeast, Northern Ireland, Northwest, Scotland, Southeast, Southwest, West Midlands, Yorkshire-Humber	CI5*plus*	1995-2012		1.6^d^	0.9 to 2.3
USA Black	SEER^f^ (9 registries): Atlanta, Connecticut, Detroit, Hawaii, Iowa, New Mexico, San Francisco-Oakland, Seattle-Puget Sound, and Utah	CI5*plus*	1978-2012		–0.8^c^	–1.4 to –0.3
USA White	SEER (9 Registries): Atlanta, Connecticut, Detroit, Hawaii, Iowa, New Mexico, San Francisco-Oakland, Seattle-Puget Sound, and Utah	CI5*plus*	1978-2012		–1.9^c^	–3.1 to –0.6

^a^APC: annual percentage change.

^b^AAPC: average annual percentage change.

^c^CI5*plus*: Cancer Incidence in Five Continents plus.

^d^Statistically significant (*P*<.05).

^e^NORDCAN: Nordic Cancer Registries.

^f^SEER: Surveillance, Epidemiology, and End Results Program.

## Discussion

This study comprehensively describes the global pattern and incidence trend of penile cancer. We found that the higher incidence and mortality of penile cancer remain centered in developing settings, such as Southern Africa, South Asia, and South America. In examining temporal trends in incidence, we found that the ASIRs of penile cancer have increased in 15 of 40 populations, 13 of which were in Europe, and decreased in 5 populations.

Although penile cancer is a rare disease, its incidence varies greatly in different regions of the world. In this study, the highest ASIRs of penile cancer occurred in Southern Africa, especially in Eswatini (ASIR: 7.0 per 100,000) and Uganda (ASIR: 4.6 per 100,000) [46,47]. Human immunodeficiency virus (HIV) and HPV are the major public health problems in Southern Africa [48,49]. The immune system clearly plays an important role in the clearance and persistence of HPV infection and in the development of penile cancer [50]. Immunocompromised patients have a higher risk of malignant transformation of HPV lesions. Men who are HIV positive have a 2- to 3-fold increased risk for penile cancer compared with their negative counterparts [50,51]. A study of heterosexual men from Uganda showed that HPV prevalence in the penis was 90.7% among men who are HIV positive and 60.9% among men who are HIV negative [52]. The prevalence of HPV in penis could also be responsible for the high incidence of penile cancer in South American countries, such as Brazil, Colombia, and Argentina [53-56].

We found a significantly increasing trend in the ASIR of penile cancer among most European countries (Italy, the Netherlands, Croatia, Czech Republic, Slovakia, and Russia) during the study period, especially in Northern Europe (United Kingdom, Lithuania, Norway, Estonia, and Cyprus). Consistent with our results, an increasing trend in the ASIR of penile cancer was previously observed in Norway (1956-2015) [21], Netherlands (1989-2006) [57], and the UK (1979-2009) [24]. Similar to our findings, population-based studies reported a stable incidence of penile cancer in Australia during 1982-2005 and in France during 1989-2011 [23,58]. Two Finnish studies reported the decreasing trend of penile cancer ASIR in 1955-1977 and 1971-1995, respectively [59,60]. Moreover, the significantly increasing trend in the ASIR of penile cancer in Finland since 1998 has been described in this study. Our study found that the other 2 populations with a significant increase in the ASIR of penile cancer were China and Israel, which is consistent with the ASIR trend in China between 2005 and 2015, as described by Lu et al [61].

There are many reasons for the increasing trend in the ASIR of penile cancer observed in the aforesaid countries. Increased exposure of the population to HPV and decreasing rates of circumcision in children may play an important role. Childhood circumcision has a strong protective effect against penile cancer [4]. In the United Kingdom, the proportion of boys circumcised fell from 35% in the early 1930s to 6.5% by the mid-1980s; however, circumcision became much less popular after the mid-1940s [62]. The populations with an increasing trend of ASIR for penile cancer had lower rates of circumcision, except for Israel [63]. The incidence is negligible in Israel owing to the practice of religious neonatal circumcision. However, not all increasing trends can be explained by falling rates of childhood circumcision. In recent decades, there have been more immigrants from Muslim countries to European countries such as Russia, France, Norway, the Netherlands, and UK, and therefore, the number of men undergoing circumcision in some countries may rise. Smoking rates decreased substantially between 1970 and 2009 across Europe, which is unlikely to account for the increasing trends in ASIR of penile cancer [64]. The increase in HPV prevalence may explain why the incidence of some cancers that are attributed to high-risk HPV infections, such as anal cancer, cervical cancer, and oropharyngeal cancer, have risen [65-67]. Several studies have reported a strong association between HPV and a higher rate of partner change [51,68]. The significantly higher risk of HPV detection is associated with a younger age at first sexual intercourse and an increase in the number of lifelong female sexual partners. Both of these factors have changed in higher-income countries [69,70]. In the past 40 years, China’s opening to the world has brought about economic recovery, but it has also led to changes in sexual behavior, which is reflected in the increase in the incidence of sexually transmitted diseases and changes in the pattern of HIV transmission [71,72].

We found that ASIR decreased in Brazil, Canada, the United States, and most Asian countries, including India, Japan, Korea, Philippines, and Thailand. Although male HPV vaccines are available in Brazil, the United States, and Canada, vaccination would not have had sufficient time to influence the rates of penile cancer in these countries. The major determinant of male circumcision in India is religion: Muslims practice male circumcision for cultural reasons, whereas the predominantly Hindu population does not. This hinders the national promotion of circumcision and is linked to the lower popularity of circumcision [73]. The improvement of personal hygiene might be responsible for the decrease in the incidence of penile cancer in some developing countries, which tend to have a large disease burden. Studies have shown that penile cancer cases in Brazil and India were mainly concentrated in areas that have the lowest HDI [74,75]. Consistent with the study of Goodman et al [76], the decreasing trends in the ASIR of penile cancer were observed in the United States, in both Whites and Blacks, which can be explained by the increasing rate of circumcision. A national probability sample of 1410 American men aged 18-59 years suggested a steady increase in the prevalence of circumcision from a low of 31% (1932) to 85% (1965) [77]. The prevalence of circumcision in the ethnic groups mentioned in this survey was negatively correlated with the incidence of penile cancer found in our analysis: Whites (81%) have a much higher circumcision rate than Blacks (65%).

The incidence trend of penile cancer observed in this study is similar to other long-lag HPV-related cancers, such as vulvar cancer and anal cancer [78,79]. The incidence trend of the other 2 cancers may not be directly comparable with penile cancer due to the different attributable risks. However, the increasing trend in some high-income countries is consistent, such as UK, Italy, and the Netherlands [78,79]. Currently, routine HPV vaccination of boys and men is implemented in several countries, such as Australia, Canada, the United States, and Austria [80]. Vaccination of boys and men may further reduce the incidence of penile cancer, anal cancer, and head and neck cancer; additionally, it may reduce the incidence of cervical cancer and its precursors by herd protection [81]. Expanding the benefits of HPV vaccination to boys and men in countries with a high burden of HPV infection should be evaluated as soon as possible.

The results in our study are enhanced by using 3 data sources (GLOBOCAN, CI5*plus*, and NORDCAN) that include the most recent data possible. Nonetheless, several limitations should be noted for this study. First, the estimates of incidence and mortality of penile cancer were obtained from GLOBOCAN, which is based on the best available data; however, in countries where the estimations are based on potentially biased, insufficient, and proxy data, the estimates should be interpreted with caution. Second, although the data for incidence trend analysis were extracted from a high-quality database (CI5*plus*), some regional data may not be representative of the entire country. Third, the analysis of data from several countries was based on small numbers, subject to substantial random variation, because of the rarity of the disease. Because of insufficient statistical power, we were unable to detect significant trends in smaller populations. Fourth, we were not able to describe trends in incidence by histological subtype or morphologic variant, nor perform age-period-cohort analysis in terms of risk factors associated with penile cancer due to data unavailability. This study could not demonstrate causality in the ASIR trend of penile cancer. Notwithstanding these weaknesses, these data are the largest currently available and allow comparisons across countries because of the uniform approach applied.

Further research is, however, needed to explain the observed regional differences. As a large proportion of penile cancer is attributable to HPV, the efficacy of HPV vaccines in high-risk groups should be assessed as soon as possible. Future research should also continue to explore the association of risk factors with prognosis in patients with penile cancer and to follow the evolution of incidence and survival of this cancer.

In conclusion, this study provides a comprehensive update on the global patterns and trends in the incidence of penile cancer. While the higher incidence and mortality of penile cancer remain in some developing countries, these have significantly increased in most European populations studied, but have also decreased in a few countries. Although there are many causes of penile cancer, HPV infection, poor penile hygiene, and lack of circumcision may play important roles. Improving penile hygiene and promoting the widespread use of male HPV vaccines should be part of prevention programs for penile cancer in the future.

## Appendix

Multimedia Appendix 1 Estimated new cases number and age-standardized incidence rates for penile cancer.

Multimedia Appendix 2 Estimated death cases number and age-standardized mortality rates for penile cancer.

## Abbreviations

AAPC: average annual percentage change
APC: annual percentage change
ASIR: age-standardized incidence rate
ASMR: age-standardized mortality rate
CI5: Cancer Incidence in Five Continents
GLOBOCAN: Global Cancer Registries
HDI: Human Development Index
HIV: human immunodeficiency virus
HPV: human papillomavirus
IARC: International Agency for Research on Cancer
LOWESS: locally weighted regression
NORDCAN: Nordic Cancer Registries
SEER: Surveillance, Epidemiology, and End Results Program

## Data Availability Statement

The data that support the findings of this study are available from the corresponding author, HZ, upon reasonable request.
