# Supplementary material for: Global Pattern and Trends in Penile Cancer Incidence: Population-Based Study
Source: JMIR Public Health Surveill. 2022 Jul 6;8(7):e34874. doi: 10.2196/34874 (PMC9301560; doi:10.2196/34874)
Supplement: Multimedia Appendix 1 [file publichealth_v8i7e34874_app1.docx]

**Multimedia Appendix 1. Estimated new cases number and age-standardized incidence rates for penile cancer.**

| **Population** | **Number** | **Uncertainty interval** | **Crude Rate** | **ASR (World)** |
| --- | --- | --- | --- | --- |
| **World** | 36068 | [30962.6-42015.2] | 0.92 | 0.80 |
| **Income levels** |  |  |  |  |
| Low middle income | 14499 | [11772.0-17857.7] | 0.94 | 1.20 |
| Upper middle income | 12228 | [10593.4-14114.9] | 0.83 | 0.65 |
| High income | 8293 | [7573.0-9081.5] | 1.40 | 0.66 |
| Low income | 1035 | [512.8-2088.8] | 0.34 | 0.64 |
| **Continents** |  |  |  |  |
| Asia | 20315 | [17288.8-23870.9] | 0.86 | 0.74 |
| Europe | 6762 | [6053.1-7553.9] | 1.90 | 0.94 |
| Latin America and the Caribbean | 4988 | [4039.3-6159.5] | 1.60 | 1.30 |
| Africa | 2060 | [1012.6-4190.6] | 0.31 | 0.53 |
| Northern America | 1741 | [1651.8-1835.1] | 0.95 | 0.51 |
| Oceania | 202 | [158.1-258.1] | 0.95 | 0.64 |
| **Countries** |  |  |  |  |
| India | 10677 | [9217.9-12367.0] | 1.50 | 1.60 |
| China | 4628 | [4092.4-5233.7] | 0.62 | 0.42 |
| Brazil | 1658 | [1417.1-1939.9] | 1.60 | 1.30 |
| United States of America | 1515 | [1338.7-1714.5] | 0.92 | 0.50 |
| Germany | 1046 | [890.7-1228.4] | 2.50 | 1.10 |
| Indonesia | 1017 | [561.1-1843.4] | 0.74 | 0.74 |
| United Kingdom | 763 | [599.9-970.5] | 2.30 | 1.20 |
| Russian Federation | 713 | [642.1-791.7] | 1.10 | 0.71 |
| Mexico | 696 | [549.7-881.2] | 1.10 | 1.00 |
| Thailand | 691 | [540.4-883.6] | 2.00 | 1.30 |
| Bangladesh | 640 | [99.1-4135.0] | 0.77 | 0.82 |
| France | 569 | [399.4-810.6] | 1.80 | 0.76 |
| Colombia | 550 | [381.7-792.5] | 2.20 | 1.90 |
| Italy | 540 | [375.8-776.0] | 1.80 | 0.79 |
| Japan | 512 | [397.5-659.6] | 0.83 | 0.25 |
| Spain | 506 | [353.3-724.7] | 2.20 | 0.95 |
| Poland | 440 | [313.7-617.2] | 2.40 | 1.30 |
| Argentina | 407 | [302.2-548.1] | 1.80 | 1.60 |
| Viet Nam | 397 | [277.1-568.8] | 0.82 | 0.76 |
| Uganda | 345 | [222.3-535.5] | 1.50 | 4.60 |
| Myanmar | 339 | [120.9-950.3] | 1.30 | 1.50 |
| Pakistan | 333 | [27.6-4017.6] | 0.29 | 0.41 |
| Ukraine | 304 | [248.1-372.5] | 1.50 | 0.94 |
| Peru | 285 | [203.7-398.8] | 1.70 | 1.40 |
| Venezuela, Bolivarian Republic of | 270 | [160.7-453.6] | 1.90 | 1.80 |
| Canada | 226 | [152.1-335.7] | 1.20 | 0.57 |
| South Africa | 211 | [176.6-252.2] | 0.72 | 0.84 |
| Romania | 196 | [137.7-279.0] | 2.10 | 1.20 |
| The Netherlands | 192 | [149.9-245.8] | 2.20 | 0.97 |
| Nepal | 185 | [112.1-305.3] | 1.40 | 1.70 |
| Angola | 170 | [133.4-216.6] | 1.00 | 2.10 |
| Bolivia, Plurinational State of | 159 | [82.3-307.1] | 2.70 | 2.00 |
| Congo, Democratic Republic of | 156 | [118.6-205.1] | 0.35 | 0.71 |
| Cuba | 154 | [108.9-217.9] | 2.70 | 1.40 |
| Mozambique | 137 | [63.4-296.0] | 0.90 | 1.80 |
| Malawi | 132 | [53.1-328.1] | 1.40 | 2.50 |
| Tanzania, United Republic of | 131 | [61.6-278.6] | 0.44 | 1.00 |
| Australia | 131 | [100.2-171.2] | 1.00 | 0.56 |
| Philippines | 126 | [67.1-236.5] | 0.23 | 0.28 |
| Zambia | 126 | [69.2-229.5] | 1.40 | 3.00 |
| Czechia | 125 | [78.3-199.7] | 2.40 | 1.10 |
| Belgium | 120 | [65.9-218.6] | 2.10 | 1.00 |
| Portugal | 119 | [74.3-190.5] | 2.50 | 1.10 |
| Paraguay | 115 | [72.5-182.4] | 3.20 | 3.40 |
| Sri Lanka | 114 | [90.0-144.4] | 1.10 | 0.86 |
| Ecuador | 113 | [69.5-183.6] | 1.30 | 1.20 |
| Sweden | 108 | [70.5-165.6] | 2.10 | 0.91 |
| Zimbabwe | 101 | [62.5-163.1] | 1.40 | 3.30 |
| Dominican Republic | 101 | [58.9-173.3] | 1.90 | 1.80 |
| Rwanda | 100 | [37.2-269.1] | 1.60 | 3.40 |
| Korea, Republic of | 98 | [67.6-142.1] | 0.38 | 0.20 |
| Greece | 97 | [61.0-154.2] | 1.90 | 0.77 |
| Chile | 93 | [59.7-145.0] | 0.99 | 0.67 |
| Cambodia | 87 | [31.0-243.9] | 1.10 | 1.40 |
| Serbia | 83 | [51.1-134.9] | 1.90 | 1.00 |
| Austria | 82 | [61.2-109.9] | 1.80 | 0.86 |
| Switzerland | 80 | [40.3-159.0] | 1.90 | 0.79 |
| Denmark | 77 | [60.2-98.5] | 2.70 | 1.20 |
| Hungary | 75 | [42.6-131.9] | 1.60 | 0.86 |
| Honduras | 70 | [28.4-172.4] | 1.40 | 1.90 |
| Norway | 66 | [50.7-85.9] | 2.40 | 1.30 |
| Malaysia | 66 | [41.4-105.3] | 0.40 | 0.39 |
| Burundi | 66 | [6.6-660.2] | 1.10 | 3.00 |
| Belarus | 55 | [40.8-74.1] | 1.30 | 0.83 |
| Bulgaria | 53 | [39.9-70.4] | 1.60 | 0.77 |
| Haiti | 52 | [30.3-89.2] | 0.92 | 1.20 |
| Korea, Democratic Republic of | 51 | [12.5-207.7] | 0.40 | 0.35 |
| Slovakia | 49 | [34.5-69.5] | 1.80 | 1.10 |
| Iran, Islamic Republic of | 47 | [29.5-74.8] | 0.11 | 0.11 |
| Kenya | 47 | [13.4-164.9] | 0.18 | 0.45 |
| Papua New Guinea | 43 | [19.3-95.9] | 0.94 | 1.60 |
| Finland | 40 | [28.7-55.8] | 1.50 | 0.68 |
| Ireland | 39 | [26.1-58.4] | 1.60 | 0.92 |
| Lao People's Democratic Republic | 38 | [13.6-106.5] | 1.00 | 1.40 |
| Costa Rica | 37 | [24.2-56.6] | 1.50 | 1.10 |
| Kazakhstan | 35 | [23.3-52.5] | 0.38 | 0.42 |
| Botswana | 35 | [22.4-54.7] | 3.10 | 4.40 |
| Nicaragua | 34 | [17.5-65.9] | 1.00 | 1.30 |
| Ethiopia | 34 | [3.4-343.1] | 0.06 | 0.09 |
| North Macedonia | 33 | [14.3-76.2] | 3.20 | 1.70 |
| Croatia | 33 | [22.7-48.0] | 1.70 | 0.82 |
| Puerto Rico | 31 | [22.4-42.9] | 2.30 | 1.10 |
| Singapore | 30 | [12.5-72.2] | 0.98 | 0.56 |
| Uruguay | 29 | [19.5-43.2] | 1.70 | 1.10 |
| South Sudan | 29 | [2.9-290.1] | 0.52 | 1.00 |
| Republic of Moldova | 28 | [12.3-63.8] | 1.40 | 1.10 |
| El Salvador | 27 | [11.7-62.1] | 0.89 | 0.84 |
| Lithuania | 26 | [16.9-40.0] | 2.10 | 1.00 |
| Panama | 25 | [11.1-56.5] | 1.20 | 0.98 |
| Turkey | 23 | [2.0-270.1] | 0.06 | 0.05 |
| Eswatini | 23 | [14.1-37.6] | 4.00 | 7.00 |
| Lesotho | 23 | [12.5-42.4] | 2.20 | 3.10 |
| Georgia | 22 | [14.0-34.5] | 1.20 | 0.68 |
| New Zealand | 21 | [12.0-36.7] | 0.89 | 0.48 |
| Ghana | 20 | [1.8-223.0] | 0.13 | 0.21 |
| Nigeria | 20 | [1.8-217.4] | 0.02 | 0.04 |
| Sudan | 19 | [6.2-58.6] | 0.09 | 0.15 |
| Latvia | 19 | [11.3-31.8] | 2.20 | 1.30 |
| Guatemala | 19 | [7.3-49.5] | 0.22 | 0.29 |
| Namibia | 19 | [11.6-31.1] | 1.50 | 2.40 |
| Jamaica | 17 | [6.3-45.7] | 1.20 | 0.93 |
| Bosnia and Herzegovina | 15 | [4.9-46.0] | 0.93 | 0.48 |
| Slovenia | 15 | [7.6-29.4] | 1.40 | 0.67 |
| Cyprus | 12 | [5.1-28.2] | 2.00 | 1.10 |
| Morocco | 12 | [1.1-130.8] | 0.07 | 0.07 |
| Afghanistan | 11 | [1.7-71.1] | 0.06 | 0.12 |
| Estonia | 11 | [5.9-20.5] | 1.80 | 0.94 |
| Togo | 10 | [1.9-54.0] | 0.24 | 0.80 |
| Kyrgyzstan | 10 | [1.7-58.9] | 0.31 | 0.37 |
| Cameroon | 10 | [0.80-122.6] | 0.08 | 0.14 |
| Egypt | 10 | [0.90-108.9] | 0.02 | 0.03 |
| Albania | 10 | [2.4-42.1] | 0.68 | 0.37 |
| Madagascar | 10 | [1.0-100.0] | 0.07 | 0.21 |
| Lebanon | 9 | [3.9-20.7] | 0.26 | 0.25 |
| Luxembourg | 9 | [3.0-26.6] | 2.80 | 1.60 |
| Trinidad and Tobago | 9 | [1.4-57.5] | 1.30 | 0.95 |
| Guinea | 8 | [0.60-109.1] | 0.13 | 0.39 |
| Mali | 8 | [0.80-84.8] | 0.08 | 0.16 |
| Israel | 7 | [3.0-16.2] | 0.16 | 0.14 |
| Armenia | 7 | [4.5-11.0] | 0.50 | 0.36 |
| Uzbekistan | 7 | [2.0-24.7] | 0.04 | 0.06 |
| Tunisia | 6 | [1.1-33.7] | 0.10 | 0.10 |
| Azerbaijan | 6 | [0.60-60.6] | 0.12 | 0.13 |
| Iceland | 5 | [1.8-13.9] | 2.90 | 1.70 |
| Montenegro | 5 | [1.8-13.6] | 1.60 | 0.82 |
| Syrian Arab Republic | 5 | [0.60-40.3] | 0.06 | 0.09 |
| Fiji | 5 | [2.4-10.5] | 1.10 | 1.40 |
| Somalia | 5 | [0.50-50.0] | 0.06 | 0.15 |
| Saint Lucia | 5 | [0.40-64.9] | 5.50 | 3.90 |
| Senegal | 5 | [0.70-38.3] | 0.06 | 0.11 |
| France, Martinique | 4 | [1.2-13.3] | 2.30 | 0.73 |
| Saudi Arabia | 4 | [0.70-22.9] | 0.02 | 0.03 |
| Guyana | 4 | [0.40-45.6] | 1.00 | 1.00 |
| Mauritius | 4 | [1.4-11.4] | 0.64 | 0.42 |
| France, Guadeloupe | 4 | [1.3-12.2] | 2.20 | 0.82 |
| Côte d'Ivoire | 4 | [0.40-41.3] | 0.03 | 0.06 |
| Algeria | 4 | [0.40-41.5] | 0.02 | 0.02 |
| Suriname | 4 | [0.50-34.3] | 1.40 | 1.50 |
| Bhutan | 4 | [2.7-6.0] | 0.98 | 0.98 |
| Cabo Verde | 3 | [0.50-16.7] | 1.10 | 1.90 |
| Chad | 3 | [2.3-3.9] | 0.04 | 0.08 |
| Malta | 3 | [0.80-11.1] | 1.40 | 0.38 |
| Burkina Faso | 3 | [0.50-16.8] | 0.03 | 0.08 |
| Mongolia | 3 | [0.90-10.1] | 0.19 | 0.21 |
| Turkmenistan | 2 | [1.2-3.3] | 0.07 | 0.07 |
| France, La Réunion | 2 | [0.30-13.4] | 0.46 | 0.22 |
| The Republic of the Gambia | 2 | [0.20-22.4] | 0.17 | 0.29 |
| Liberia | 2 | [0.30-15.3] | 0.08 | 0.15 |
| Bahamas | 2 | [1.7-2.4] | 1.00 | 0.72 |
| Solomon Islands | 2 | [0.90-4.5] | 0.57 | 0.89 |
| United Arab Emirates | 2 | [0.20-17.9] | 0.03 | 0.08 |
| Kuwait | 1 | [0.10-9.5] | 0.04 | 0.04 |
| Eritrea | 1 | [0.10-10.0] | 0.06 | 0.08 |
| Sierra Leone | 1 | [0.30-3.6] | 0.03 | 0.02 |
| Oman | 1 | [0.10-11.0] | 0.03 | 0.03 |
| Jordan | 1 | [0.10-10.7] | 0.02 | 0.04 |
| Qatar | 1 | [0.10-11.9] | 0.05 | 0.09 |
| Barbados | 1 | [0.80-1.2] | 0.72 | 0.43 |
| French Guiana | 1 | [0.20-6.4] | 0.68 | 0.80 |
| Gabon | 1 | [0.10-10.9] | 0.09 | 0.14 |
| Iraq | 1 | [0.10-9.0] | 0.00 | 0.00 |
| Central African Republic | 1 | [0.80-1.3] | 0.04 | 0.08 |
| Comoros | 1 | [0.10-10.0] | 0.23 | 0.36 |
