# Supplementary material for: Global Pattern and Trends in Penile Cancer Incidence: Population-Based Study
Source: JMIR Public Health Surveill. 2022 Jul 6;8(7):e34874. doi: 10.2196/34874 (PMC9301560; doi:10.2196/34874)
Supplement: Multimedia Appendix 2 [file publichealth_v8i7e34874_app2.docx]

**Multimedia Appendix 2. Estimated death cases number and age-standardized mortality rates for penile cancer.**

| **Population** | **Number** | **Uncertainty interval** | **Crude Rate** | **ASR (World)** |
| --- | --- | --- | --- | --- |
| World | 13211 | [10686.7-16331.5] | 0.34 | 0.29 |
| Income levels | |  |  |  |
| Low middle income | 6305 | [4009.8-9914.0] | 0.41 | 0.51 |
| Upper middle income | 4197 | [3361.1-5240.7] | 0.29 | 0.22 |
| High income | 2204 | [1876.4-2588.8] | 0.36 | 0.16 |
| Low income | 504 | [229.9-1105.0] | 0.17 | 0.32 |
| Continents | |  |  |  |
| Asia | 8189 | [5922.4-11323.0] | 0.35 | 0.30 |
| Europe | 1938 | [1569.7-2392.7] | 0.54 | 0.25 |
| Latin America and the Caribbean | 1627 | [1420.3-1863.8] | 0.51 | 0.44 |
| Africa | 942 | [420.0-2112.7] | 0.14 | 0.25 |
| Northern America | 477 | [429.6-529.6] | 0.26 | 0.13 |
| Oceania | 38 | [18.8-76.7] | 0.18 | 0.12 |
| Countries |  |  |  |  |
| India | 4760 | [4070.0-5566.9] | 0.66 | 0.72 |
| China | 1565 | [1214.2-2017.2] | 0.21 | 0.14 |
| Brazil | 539 | [477.2-608.9] | 0.52 | 0.42 |
| United States of America | 414 | [350.2-489.4] | 0.25 | 0.13 |
| Indonesia | 347 | [189.6-635.1] | 0.25 | 0.26 |
| Russian Federation | 324 | [179.4-585.1] | 0.48 | 0.31 |
| Germany | 281 | [228.3-345.8] | 0.68 | 0.25 |
| Thailand | 258 | [189.0-352.3] | 0.76 | 0.47 |
| Bangladesh | 246 | [43.3-1396.0] | 0.30 | 0.33 |
| Mexico | 219 | [180.2-266.2] | 0.35 | 0.33 |
| Poland | 174 | [133.5-226.9] | 0.95 | 0.49 |
| Uganda | 165 | [98.9-275.2] | 0.73 | 2.40 |
| Colombia | 162 | [123.5-212.6] | 0.65 | 0.56 |
| Japan | 155 | [123.3-194.9] | 0.25 | 0.07 |
| Myanmar | 152 | [63.7-362.7] | 0.58 | 0.72 |
| United Kingdom | 150 | [117.5-191.5] | 0.45 | 0.19 |
| Argentina | 148 | [117.2-186.9] | 0.67 | 0.53 |
| Viet Nam | 147 | [99.0-218.4] | 0.30 | 0.28 |
| Italy | 144 | [109.7-189.1] | 0.49 | 0.17 |
| France | 136 | [103.7-178.4] | 0.43 | 0.17 |
| Spain | 119 | [90.6-156.3] | 0.52 | 0.21 |
| Pakistan | 116 | [69.4-194.0] | 0.10 | 0.14 |
| Nepal | 111 | [62.2-198.0] | 0.83 | 0.99 |
| Ukraine | 89 | [66.4-119.3] | 0.44 | 0.27 |
| Venezuela, Bolivarian Republic of | 88 | [59.6-130.0] | 0.63 | 0.60 |
| Peru | 80 | [60.4-106.0] | 0.49 | 0.41 |
| Congo, Democratic Republic of | 76 | [45.0-128.3] | 0.17 | 0.36 |
| Angola | 73 | [43.3-123.2] | 0.45 | 0.92 |
| Mozambique | 72 | [29.4-176.4] | 0.47 | 1.00 |
| South Africa | 71 | [50.7-99.4] | 0.24 | 0.31 |
| Canada | 63 | [47.7-83.1] | 0.34 | 0.14 |
| Malawi | 62 | [22.7-169.3] | 0.66 | 1.20 |
| Tanzania, United Republic of | 61 | [24.9-149.5] | 0.20 | 0.48 |
| Zambia | 58 | [28.5-118.2] | 0.64 | 1.30 |
| Romania | 58 | [43.8-76.8] | 0.62 | 0.32 |
| Cuba | 57 | [42.7-76.2] | 1.00 | 0.49 |
| Bolivia, Plurinational State of | 53 | [28.4-98.9] | 0.90 | 0.69 |
| Sri Lanka | 50 | [37.2-67.3] | 0.49 | 0.37 |
| Zimbabwe | 46 | [26.2-80.8] | 0.65 | 1.70 |
| Rwanda | 46 | [14.4-147.4] | 0.72 | 1.70 |
| Philippines | 45 | [22.2-91.3] | 0.08 | 0.11 |
| Chile | 41 | [28.8-58.4] | 0.43 | 0.28 |
| Cambodia | 39 | [16.3-93.1] | 0.48 | 0.72 |
| Paraguay | 38 | [25.8-55.9] | 1.00 | 1.20 |
| Ecuador | 36 | [24.1-53.8] | 0.41 | 0.40 |
| Serbia | 36 | [24.3-53.4] | 0.84 | 0.42 |
| The Netherlands | 35 | [24.3-50.4] | 0.41 | 0.16 |
| Greece | 35 | [23.7-51.6] | 0.68 | 0.25 |
| Portugal | 34 | [23.3-49.6] | 0.70 | 0.25 |
| Burundi | 34 | [3.3-348.9] | 0.58 | 1.70 |
| Czechia | 34 | [23.5-49.2] | 0.64 | 0.29 |
| Dominican Republic | 32 | [19.8-51.7] | 0.59 | 0.57 |
| Sweden | 30 | [19.2-46.9] | 0.59 | 0.24 |
| Honduras | 25 | [14.1-44.4] | 0.51 | 0.70 |
| Malaysia | 23 | [13.6-38.8] | 0.14 | 0.14 |
| Haiti | 23 | [14.2-37.1] | 0.41 | 0.55 |
| Iran, Islamic Republic of | 22 | [13.1-37.0] | 0.05 | 0.05 |
| Hungary | 22 | [14.2-34.2] | 0.48 | 0.25 |
| Korea, Republic of | 22 | [13.2-36.6] | 0.09 | 0.04 |
| Australia | 21 | [12.9-34.2] | 0.17 | 0.07 |
| Kenya | 21 | [4.7-93.3] | 0.08 | 0.19 |
| Belgium | 20 | [12.1-33.1] | 0.35 | 0.15 |
| Denmark | 20 | [11.9-33.6] | 0.69 | 0.27 |
| Korea, Democratic Republic of | 20 | [3.0-135.3] | 0.16 | 0.13 |
| Austria | 20 | [12.2-32.8] | 0.45 | 0.19 |
| Belarus | 18 | [10.5-30.8] | 0.41 | 0.23 |
| Switzerland | 18 | [10.5-31.0] | 0.42 | 0.17 |
| Lao People's Democratic Republic | 17 | [7.1-40.6] | 0.47 | 0.65 |
| Ethiopia | 17 | [1.2-250.1] | 0.03 | 0.05 |
| Nicaragua | 16 | [9.0-28.5] | 0.49 | 0.66 |
| Bulgaria | 16 | [9.4-27.2] | 0.47 | 0.22 |
| Slovakia | 16 | [9.1-28.1] | 0.60 | 0.34 |
| South Sudan | 15 | [1.5-153.9] | 0.27 | 0.51 |
| Papua New Guinea | 13 | [0.90-180.4] | 0.28 | 0.47 |
| Croatia | 13 | [7.0-24.2] | 0.66 | 0.28 |
| Sudan | 12 | [3.6-39.9] | 0.05 | 0.10 |
| North Macedonia | 12 | [6.0-24.0] | 1.20 | 0.62 |
| Panama | 12 | [5.9-24.2] | 0.56 | 0.49 |
| Kazakhstan | 12 | [6.0-23.9] | 0.13 | 0.14 |
| Botswana | 12 | [7.1-20.3] | 1.10 | 1.50 |
| Georgia | 11 | [5.8-20.7] | 0.58 | 0.33 |
| Ireland | 11 | [5.6-21.6] | 0.45 | 0.23 |
| Republic of Moldova | 11 | [5.8-21.0] | 0.57 | 0.42 |
| Lithuania | 11 | [5.8-20.9] | 0.87 | 0.41 |
| Lesotho | 11 | [5.9-20.5] | 1.00 | 1.70 |
| Costa Rica | 11 | [5.3-22.7] | 0.43 | 0.34 |
| Norway | 10 | [4.9-20.4] | 0.36 | 0.19 |
| Uruguay | 10 | [5.0-20.0] | 0.60 | 0.31 |
| Finland | 10 | [5.0-20.1] | 0.37 | 0.15 |
| Eswatini | 10 | [5.4-18.6] | 1.80 | 3.50 |
| El Salvador | 9 | [4.2-19.2] | 0.30 | 0.26 |
| Nigeria | 9 | [5.3-15.2] | 0.01 | 0.02 |
| Morocco | 9 | [0.60-132.4] | 0.05 | 0.05 |
| Ghana | 8 | [0.50-117.7] | 0.05 | 0.09 |
| Puerto Rico | 8 | [4.1-15.8] | 0.59 | 0.31 |
| Guatemala | 8 | [3.5-18.4] | 0.09 | 0.13 |
| Bosnia and Herzegovina | 7 | [2.7-18.0] | 0.44 | 0.20 |
| Turkey | 7 | [2.1-23.3] | 0.02 | 0.02 |
| Namibia | 7 | [3.7-13.1] | 0.57 | 0.86 |
| Jamaica | 6 | [2.6-14.1] | 0.41 | 0.30 |
| Latvia | 6 | [2.6-13.8] | 0.69 | 0.36 |
| Togo | 6 | [1.1-31.8] | 0.15 | 0.37 |
| Albania | 6 | [1.4-25.3] | 0.41 | 0.22 |
| Singapore | 6 | [1.9-19.2] | 0.20 | 0.11 |
| Lebanon | 5 | [1.7-14.3] | 0.15 | 0.13 |
| Afghanistan | 5 | [0.90-28.4] | 0.03 | 0.06 |
| Madagascar | 5 | [0.50-51.3] | 0.04 | 0.12 |
| Montenegro | 4 | [1.3-12.6] | 1.30 | 0.77 |
| New Zealand | 4 | [0.80-18.9] | 0.17 | 0.08 |
| Guinea | 4 | [0.30-58.8] | 0.06 | 0.17 |
| Mali | 4 | [0.30-58.8] | 0.04 | 0.06 |
| Armenia | 4 | [1.5-10.4] | 0.29 | 0.18 |
| Cameroon | 4 | [2.4-6.8] | 0.03 | 0.06 |
| Egypt | 4 | [0.30-58.9] | 0.01 | 0.01 |
| Estonia | 4 | [1.2-13.6] | 0.64 | 0.24 |
| Syrian Arab Republic | 3 | [0.40-22.5] | 0.03 | 0.05 |
| Somalia | 3 | [0.30-30.8] | 0.04 | 0.09 |
| Kyrgyzstan | 3 | [0.70-13.2] | 0.09 | 0.10 |
| Suriname | 3 | [0.50-19.8] | 1.00 | 1.10 |
| Uzbekistan | 3 | [1.0-8.8] | 0.02 | 0.02 |
| Slovenia | 3 | [1.0-8.7] | 0.29 | 0.11 |
| Israel | 2 | [0.50-7.3] | 0.05 | 0.03 |
| Chad | 2 | [1.2-3.4] | 0.02 | 0.06 |
| Saudi Arabia | 2 | [0.30-15.0] | 0.01 | 0.01 |
| Côte d'Ivoire | 2 | [1.2-3.4] | 0.02 | 0.03 |
| Burkina Faso | 2 | [0.30-13.4] | 0.02 | 0.07 |
| Algeria | 2 | [0.10-29.4] | 0.01 | 0.01 |
| Tunisia | 2 | [0.30-13.4] | 0.03 | 0.04 |
| Senegal | 2 | [0.30-13.4] | 0.02 | 0.05 |
| Azerbaijan | 2 | [0.10-31.8] | 0.04 | 0.05 |
| Turkmenistan | 1 | [0.20-4.8] | 0.03 | 0.04 |
| France, Martinique | 1 | [0.10-9.7] | 0.58 | 0.12 |
| Cyprus | 1 | [0.20-5.3] | 0.17 | 0.13 |
| France, La Réunion | 1 | [0.10-14.3] | 0.23 | 0.12 |
| The Republic of the Gambia | 1 | [0.10-14.7] | 0.08 | 0.16 |
| Liberia | 1 | [0.10-6.7] | 0.04 | 0.08 |
| Sierra Leone | 1 | [0.20-4.1] | 0.03 | 0.02 |
| Bahamas | 1 | [0.10-10.4] | 0.52 | 0.36 |
| Bhutan | 1 | [0.70-1.5] | 0.24 | 0.30 |
| Mongolia | 1 | [0.20-4.9] | 0.06 | 0.09 |
| Central African Republic | 1 | [0.60-1.7] | 0.04 | 0.08 |
| United Arab Emirates | 1 | [0.10-17.2] | 0.01 | 0.07 |
